# Behavioural and physiological adaptations to low-temperature environments in the common frog, *Rana temporaria*

**DOI:** 10.1186/1471-2148-14-110

**Published:** 2014-05-23

**Authors:** Anna P Muir, Roman Biek, Barbara K Mable

**Affiliations:** 1 Institute of Biodiversity, Animal Health and Comparative Medicine, University of Glasgow, Glasgow G12 8QQ, UK

**Keywords:** Routine metabolic rate, Freeze tolerance, Spawning temperature, Altitude, Scotland

## Abstract

**Background:**

Extreme environments can impose strong ecological and evolutionary pressures at a local level. Ectotherms are particularly sensitive to low-temperature environments, which can result in a reduced activity period, slowed physiological processes and increased exposure to sub-zero temperatures. The aim of this study was to assess the behavioural and physiological responses that facilitate survival in low-temperature environments. In particular, we asked: 1) do high-altitude common frog (*Rana temporaria*) adults extend the time available for larval growth by breeding at lower temperatures than low-altitude individuals?; and 2) do tadpoles sampled from high-altitude sites differ physiologically from those from low-altitude sites, in terms of routine metabolic rate (RMR) and freeze tolerance? Breeding date was assessed as the first day of spawn observation and local temperature recorded for five, paired high- and low-altitude *R. temporaria* breeding sites in Scotland. Spawn was collected and tadpoles raised in a common laboratory environment, where RMR was measured as oxygen consumed using a closed respiratory tube system. Freeze tolerance was measured as survival following slow cooling to the point when all container water had frozen.

**Results:**

We found that breeding did not occur below 5°C at any site and there was no significant relationship between breeding temperature and altitude, leading to a delay in spawning of five days for every 100 m increase in altitude. The relationship between altitude and RMR varied by mountain but was lower for individuals sampled from high- than low-altitude sites within the three mountains with the highest high-altitude sites (≥900 m). In contrast, individuals sampled from low-altitudes survived freezing significantly better than those from high-altitudes, across all mountains.

**Conclusions:**

Our results suggest that adults at high-altitude do not show behavioural adaptations in terms of breeding at lower temperatures. However, tadpoles appear to have the potential to adapt physiologically to surviving at high-altitude via reduced RMR but without an increase in freeze tolerance. Therefore, survival at high-altitude may be facilitated by physiological mechanisms that permit faster growth rates, allowing completion of larval development within a shorter time period, alleviating the need for adaptations that extend the time available for larval growth.

## Background

Stressful environments (environments outside the optimum conditions for a particular species) can impose strong ecological and evolutionary pressures at a local level [1,2]. Population persistence depends on the ability of individuals to respond to environmental stress through adaptive, plastic or behavioural mechanisms that maximise fitness [3]. Extremes of pH (common frog; [4]), water availability (wild mustard [5]), and temperature (redband trout; [2]) have been observed to drive adaptive population divergence. High-latitudes and altitudes experience low temperatures that can result in shorter activity periods and longer periods of freezing [3,6,7]. Plastic and adaptive responses to low temperature environments have been widely recorded (for a review see [8]) and can result in cryptic divergence between populations inhabiting different temperature regimes (counter-gradient variation; [9]). Temperature is often the major abiotic factor that influences physiological mechanisms in ectotherms [10,11] and growth slows in response to cold environments [12]. Reduced activity periods in low-temperature environments, in combination with low-temperature driven growth-rate reductions, can result in lower sizes at important life-history events such as metamorphosis and reproduction [13]. Smaller sizes can translate to lower fitness when weight is positively correlated with survival or reproductive success [13,14].

Assessing the mechanisms that facilitate survival in challenging environments is important for understanding how populations respond to ecological and evolutionary pressures, particularly in a globally changing climate [15]. Potential responses to maximise size at important life history stages in low-temperature environments include altering metabolic rate (e.g. to allow more resources to be allocated to growth; [6,16]), developmental period (e.g. delaying sexual maturity; [17-19]), or temperature activity range (e.g. breeding at lower temperatures; [20]). Populations that inhabit high-altitude environments experience lower temperatures and shorter activity periods than their low-altitude neighbours and offer an excellent opportunity to assess how survival is facilitated in environments where growth is constrained [21]. Amphibians are a particularly good model for studying physiological and behavioural responses to growth constraints, as size at metamorphosis is positively correlated with survival in the subsequent terrestrial life-history stages [22,23].

Variation in metabolic rates between individuals is a common occurrence in nature [24], but the effects on fitness are still relatively unknown [25]. Resting metabolic rate, here defined as the energetic cost of self-maintenance [26], has been linked to multiple physiological and behavioural traits including predator avoidance, foraging behaviour, swimming performance and growth [13,24]. Growth imposes a significant physiological cost and can result in a trade-off with other physiological mechanisms [13,27,28], especially when resources are limited [29]. As the vast majority of energy expenditure in ectotherms is maintenance costs (80-85%), small differences in resting metabolic rate can result in large differences in energy available for growth [29]. An increased growth rate can result in a larger size at important life history events and has been linked to a reduced resting metabolic rate in sagebrush lizards at high-altitude [6], Sydney rock oysters from growth rate-selected stock [30] and snapping turtles [31]. Attempts to assess the physiological trade-offs facilitating higher growth rates in larval common frog (*Rana temporaria*) at high latitudes have found no link to reduced metabolic rates [16]. However, as temperature is not linearly related to latitude in Sweden [7], where these experiments were conducted, these results may mask the true nature of the temperature-metabolic rate relationship. Therefore, further research in a system with a linear temperature change is required to elucidate the relationship between resting metabolic rate, growth rate and temperature.

Another potential response to maximise size at important life-history events, is to increase the time available for growth prior to metamorphosis or reproduction by extending development over multiple growth periods [18]. The concept of delayed development, or diapause, has been commonly observed in insects, often in terms of cohort splitting where different cohorts within a population complete development at different times of the year, or even in different years [18]. In amphibians, the period immediately prior to metamorphic climax is accompanied by a loss of weight [32], but a lower weight decreases the chances of adult survival [22]. Therefore, overwintering at a higher weight, but still at the larval stage, and metamorphosing the following year has the potential to increase survival, and has been recorded in a number of temperate amphibian species [19,32]. However, low winter temperatures at high-altitude can lead to prolonged periods of freezing [33]. Therefore, in order to survive, overwintering larval amphibians must be able to respond to freezing temperatures via freeze avoidance (i.e. inhabiting environments that buffer individuals from freezing temperatures) or freeze tolerance (survival of extensive freezing of body fluids; [3,34,35]). Freeze tolerance depends on the ability to restrict ice formation to extra-cellular areas, which is mediated by accumulation of low molecular weight carbohydrates in the blood [3,34]. The ability to tolerate freezing has been linked to glucose accumulation in the blood, via release of liver glycogen, in the frogs *R. sylvatica, R. lessonae* and *R. esculenta*[3,34], and with glycerol accumulation in *Hyla versicolor*[36]. However, all previous studies have focussed on freeze tolerance in adult amphibians and the potential for freeze survival in the larval stage has, to the best of our knowledge, never been studied. The ability of a tadpole to survive freezing would extend the time available for growth to, and thus size at, metamorphosis in amphibians breeding in temperate climates.

A third alternative response to larval growth constraints would be for adults to adapt behaviourally rather than amphibian larvae adapting physiologically. Adults have the potential to expand the growing season for larvae by breeding earlier in the year [20]. In temperate amphibians, breeding is closely linked to temperature [37] and frequently occurs immediately after winter dormancy (e.g. *Bufo bufo, R. chinensis, R. sylvatica and R. temporaria;*[38,39]). By adults becoming active and breeding at lower temperatures, larvae would have longer to grow and develop prior to winter dormancy. The longer time available for growth would allow larvae to reach a larger size at metamorphosis and thus have an increased chance of survival as adults [22].

The common frog (*R. temporaria*) is the most widespread amphibian in Europe and occurs from zero to 2742 metres above sea level within its range, and to over a thousand metres on the mountains of Scotland [40-42]. It is an explosive breeder, with communal spawning taking place immediately after winter dormancy [43]; a 5°C temperature threshold is generally considered to initiate activity and spawning [44]. *R. temporaria* larvae show increased growth rates in response to low temperatures experienced at high-latitudes and altitudes throughout its range [16,45]. We have previously shown that local adaptation to high-altitude environments occurs even in the face of high gene flow, suggesting that temperature exerts a strong selective pressure [45]. However, there are also reports of *R. temporaria* overwintering as tadpoles in Scotland, although it is currently unclear whether this response is particularly linked to low-temperature environments [19]. The mountains of Scotland offer an excellent opportunity to study the responses that facilitate survival in low-temperature environments, as there is continuous habitat along altitudinal gradients, with temperature decreasing linearly by 0.65°C for every 100 m gain in altitude [40,46,47]. Individuals from high-altitude sites in Scotland experience substantially lower temperatures than their low-altitude counterparts, with an average mean annual temperature reduction of 4.5°C at high- compared to low-altitude breeding sites [40].

The overall aim of this study was to assess the physiological and behavioural responses of common frogs in Scotland that facilitate survival in low-temperature environments. In particular, this study answers the questions: 1) do high-altitude adults extend the time available for larval growth by breeding at lower temperatures than low-altitude individuals?; and 2) do tadpoles sampled from high-altitude sites differ physiologically from those from low-altitude sites, in terms of routine metabolic rate and freeze tolerance?

## Results

### Adult spawning behaviour in relation to altitude

**Figure 1:**
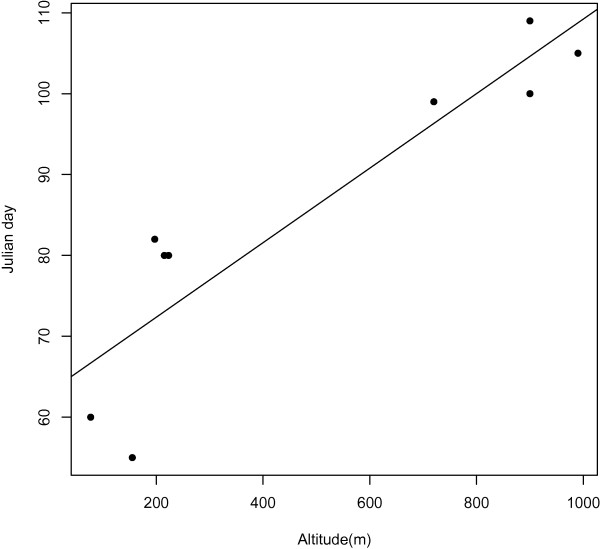
**The Julian day at which spawn was first observed by altitude, fitted with the linear regression line:** Julian day = (0.05 × Altitude) + 63.14(r ^2^ = 0.80, p < 0.01).

**Figure 2:**
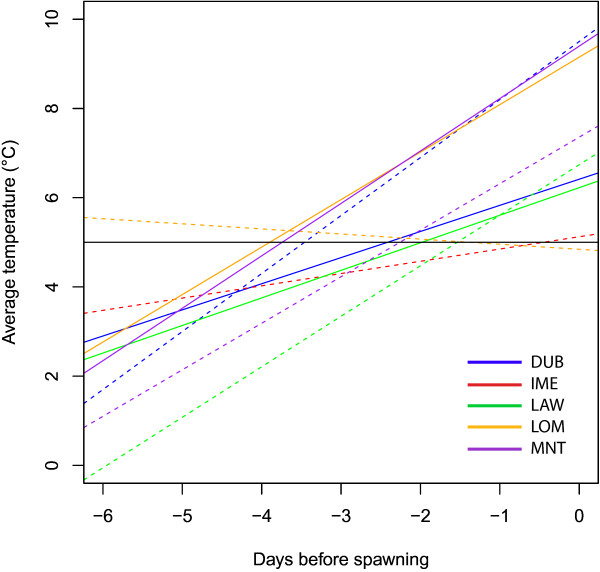
**The average daily temperature for the week prior to egg mass observation (days before spawning) for each site, seen as a linear regression line of the points.** Solid lines show high- and dashed lines show low-altitude sites per mountain. The black horizontal line shows the 5°C threshold generally considered to limit activity in *R. temporaria.*

Temperature on the day of egg mass observation was, on average, 7.5 ± 2.1°C (Table 1) and did not vary predictably with altitude (r^2^ = -0.03, p = 0.41). Likewise, no significant regression with altitude was found for the average temperature in the week prior to egg mass observation (mean temperature = 4.8 ± 0.9°C; r^2^ = -0.06, p = 0.49). Degree days prior to egg mass collection was highly variable across sites (24.5 ± 19.1; Table 1) but also did not show a significant relationship with altitude (r^2^ = 0.26, p = 0.09). The date of egg mass collection was on average 30 days later at high- compared to low-altitude sites (Table 1) and Julian spawning day showed a significant positive relationship with altitude (r^2^ = 0.80, p < 0.01): individuals spawned 5 days later for every 100 m gain in altitude (Figure 1). The daily mean temperature at all sites had exceeded the threshold value of 5°C in the week prior to egg mass collection (Figure 2).

**Table 1:** Spawning date and temperature by mountain and altitude, shown as the date of egg mass observation (observation date) and corresponding Julian day; alongside the degree days prior to egg mass observation, the daily mean temperature on the day of egg mass observation (observation day temp; °C), and the mean temperature of the week prior to egg mass observation (week prior temp; °C)

**Mountain**	**Altitude**	**Observation date**	**Julian day**	**Degree days**	**Observation day temp**	**Week prior temp**
DUB	HIGH	19-Apr	109	30.6	7.1 ± 7.1	4.7 ± 5.4
DUB	LOW	23-Mar	82	33.5	6.8 ± 4.6	5.6 ± 5.5
IME	HIGH	02-Apr	92	NA*	NA*	NA*
IME	LOW	24-Feb	55	1.5	5.8 ± 0.3	4.3 ± 0.9
LAW	HIGH	15-Apr	105	31.1	5.5 ± 2.1	4.3 ± 4.2
LAW	LOW	21-Mar	80	22.8	8.3 ± 2.6	3.3 ± 4.0
LOM	HIGH	09-Apr	99	62.9	10.8 ± 4.5	6.0 ± 3.9
LOM	LOW	01-Mar	60	5.5	4.5 ± 0.6	5.3 ± 1.1
MNT	HIGH	10-Apr	100	28.0	9.8 ± 4.0	5.9 ± 4.1
MNT	LOW	21-Mar	80	5.1	8.8 ± 2.4	4.2 ± 4.6

Observation day temp and week prior temp are mean values and are accompanied by their standard deviation.

*Data not available due to logger failure.

### Larval physiology in relation to altitude

#### Routine metabolic rate

**Figure 3:**
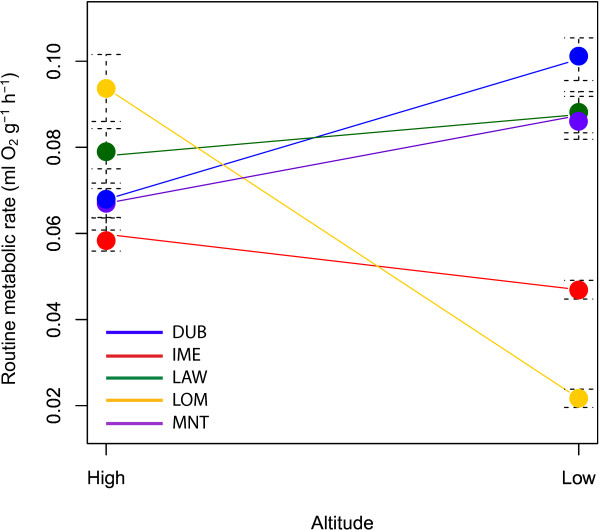
**Routine metabolic rate by mountain and altitude.** The mean routine metabolic rate per site is shown by a circle, with the bars representing the standard deviation around the mean. Low- and high- altitude sites within each mountain are linked using a straight line; a positive line shows that RMR is higher at low- vs. high-altitude, whereas a negative line shows that RMR is lower at high- vs. low-altitude.

Between eight and 20 individuals per site were measured for RMR, due to varying levels of mortality (Mean = 16 ± 5; Table 2). Mean RMR per site varied between 0.02 ml O_2_ g^-1^ h^-1^ (LOMHIGH) and 0.10 ml O_2_ g^-1^ h^-1^ (DUBLOW), with an overall average of 0.07 ± 0.02 ml O_2_ g^-1^ h^-1^ (Table 2). Mountain, altitude, and their interaction were found to be significant in predicting RMR. A Tukey’s HSD test showed a significant difference between high- and low-altitude RMR in individuals from three of the mountains: DUB (diff = 0.03, p < 0.01), MNT (diff = 0.02, p = 0.03) and LOM (diff = -0.07, p < 0.01) (Table 2). The difference between high- and low-altitude RMR was not significant for IME (diff = -0.01, p = 0.49) and LAW (diff = 0.01, p = 0.94). The direction of the relationship varied between mountains, with individuals from DUB, LAW and MNT showing a trend for lower RMR at high- compared to low-altitude, whereas individuals from IME and LOM had higher RMR at high-altitude (Figure 3). The post hoc power analysis of the ANOVA used in the Tukey’s HSD revealed an effect size of 0.29 giving an achieved power of 0.69 to determine a significant difference between the means of RMR by altitude within each mountain. The power to confidently conclude that no significant interactions have been missed (a type II error), is generally set at 0.8 [48].

**Table 2:** Physiological trait variation by mountain and altitude measured in a common environment

**Mountain**	**Altitude**	**RMR n**	**RMR (ml O**_**2 **_**g**^**-1**^ **h**^**-1**^**)**	**Tukey’s HSD Diff between means**	**Tukey’s HSD p value**	**Freeze n**	**Survival**
DUB	HIGH	8	0.07 ± 0.02			0	NA
DUB	LOW	19	0.10 ± 0.02	0.03	<0.01*	10	0.70
IME	HIGH	20	0.06 ± 0.02			10	0.20
IME	LOW	19	0.05 ± 0.01	-0.01	0.49	10	0.70
LAW	HIGH	10	0.08 ± 0.02			10	0.40
LAW	LOW	19	0.09 ± 0.02	0.01	0.94	10	0.90
LOM	HIGH	14	0.09 ± 0.03			7	0.29
LOM	LOW	12	0.02 ± 0.01	-0.07	<0.01*	10	0.90
MNT	HIGH	20	0.07 ± 0.01			10	0.40
MNT	LOW	20	0.09 ± 0.02	0.02	0.03*	10	0.90

Shown are the number of individuals per site measured (n), the mean routine metabolic rate (RMR) and the proportion of survivors following freezing (Survival). Standard deviations are indicated for RMR, whereas freeze survival is shown as a single measurement per site. The results of the Tukey’s HSD test of significant difference between the means of RMR of individuals from low- and high-altitude sites, by mountain are shown (Tukey’s HSD p value). A positive difference between the means (Diff between means) shows that individuals from high-altitude have a lower mean RMR than those from low-altitude, and a negative difference between the means shows that individuals from low-altitude have a lower RMR than those from high-altitude.

*Significant at p < 0.05.

NA: No freeze tolerance results are available for DUBHIGH due to complete tadpole mortality prior to the freeze tolerance experiment.

#### Freeze tolerance

**Figure 4:**
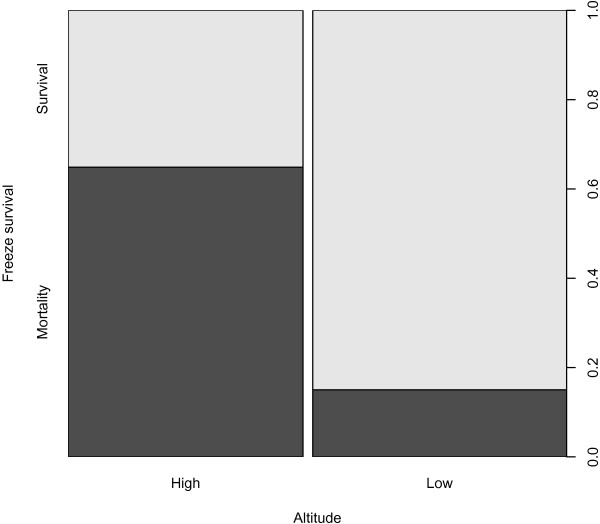
The proportion of individuals from high- versus low-altitude sites that survived freezing.

Ten individuals per site were tested for freeze tolerance, except for LOMHIGH (seven individuals) and DUBLOW (zero individuals) (Table 2), due to variable tadpole mortality prior to the experiment. Between 10% (LOMLOW, MNTLOW and LAWLOW) and 80% (IMEHIGH) mortality was observed post-freezing across sites (Mean survival = 0.60 ± 0.28; Table 2). Out of 50 tadpoles tested for freeze survival from low-altitude sites, 41 survived (82%), compared with 12/37 (32%) tadpoles from high-altitude sites (Figure 4). Altitude and weight, but not their interaction, significantly changed the log likelihood when removed from the GLMM and were thus included in the final model. There was no significant effect on the log likelihood of the model when mountain was removed from the model and thus mountain was not included in the final model (Additional file 1). The results of the GLMM using the final model (RMR ~ altitude + weight) showed that individuals from low-altitude sites had significantly higher survival than those from high-altitude sites (z = 4.20, p < 0.01).

## Discussion

### Adult spawning behaviour in relation to altitude

No significant relationship was found between spawning date and either the temperatures recorded on the day of, or in the week prior to, egg mass observation that would have suggested that breeding occurs at lower temperatures at high altitude sites. Degree days also did not show a significant relationship with altitude but they were highly variable across sites (Table 1), suggesting that degree days are not an accurate predictor of spawning activity in *R. temporaria*. All sites had exceeded the 5°C temperature threshold generally thought to initiate activity and breeding in *R. temporaria*[44] in the week prior to spawning (Figure 2). Our results therefore support 5°C as the activity threshold for *R. temporaria* regardless of altitude of breeding site, and demonstrate that high-altitude individuals experience a longer period of low-temperatures and delayed spawning compared to low-altitude individuals. The date of spawning was 5 days later for every 100 m gain in altitude (Figure 1). As mean annual temperature decreases by 0.65°C for every 100 m increase in altitude in this system [40], we can infer that spawning is on average one day earlier for every 0.65°C increase in mean annual temperature. However, further information regarding the date of spawning between 223 m and 720 m (currently not available for this system) is needed to confirm this pattern. It is interesting to note that for the mountains with the highest high-altitude sites (>900 m: DUB, LAW and MNT), the average time difference between spawning at low-and high-altitude is only 24 days, compared with 38 days observed difference between spawning at low- and high-altitude in IME and LOM (high-altitude sites at 703 m and 720 m, respectively; Tables 1 and 3). This could suggest that high-altitude individuals at the highest breeding sites are breeding at a lower temperature. However, this is not reflected as a significant difference between high- and low-altitude spawning temperatures in these mountains (Table 1). The longer period of low temperatures at high- compared to low-altitudes prior to spawning supports the hypothesis that high-altitude individuals experience a shorter annual activity period but does not support the hypothesis that breeding occurs at lower temperatures at high altitudes to provide a longer developmental period, facilitating survival at high-altitude within *R. temporaria*.

**Table 3:** Locations of study sites in Scotland including site name (study mountain and whether high- or low-altitude) with associated abbreviation, latitude, longitude and altitude (metres above sea level)

**Site**	**Abbreviation**	**Latitude**	**Longitude**	**Altitude**
Beinn Dubhchraig High	DUBHIGH	56.3951	-4.7506	900
Beinn Dubhchraig Low	DUBLOW	56.4212	-4.6945	197
Beinn Ime High	IMEHIGH	56.2347	-4.8123	703
Beinn Ime Low	IMELOW	56.2046	-4.7628	155
Ben Lawers High	LAWHIGH	56.5423	-4.2291	990
Ben Lawers Low	LAWLOW	56.5002	-4.2354	215
Ben Lomond High	LOMHIGH	56.1857	-4.6478	720
Ben Lomond Low	LOMLOW	56.1598	-4.6363	77
Meall nan Tarmachan High	MNTHIGH	56.5188	-4.2958	900
Meall nan Tarmachan Low	MNTLOW	56.4994	-4.2523	223

Breeding at a lower temperature would allow individuals to spawn earlier in the year, thus providing their offspring with a longer period in which to develop prior to metamorphosis [20], such as has been found for amphibian species at higher latitudes compared to their lower latitude counterparts [20] but, to the best of our knowledge, has not yet been observed within-species. However, phenological studies that have quantified within-species breeding temperature have typically used local weather station data [49,50] and so the spatial scale might not be not fine enough to represent local conditions in mountain areas, where temperatures can vary rapidly over short geographical distances [21,47,51]. Therefore, such studies are liable to miss within-species differences in spawning temperature in relation to altitude. When we quantified temperature at a local level, our results support the absence of a shift in breeding temperature in low-temperature environments, contributing to knowledge of the fundamental niche of *R. temporaria*. However, we did find that *R. temporaria* can substantially alter the date they breed in response to the environmental conditions experienced, even between geographically close breeding sites, demonstrating a high degree of plasticity in terms of breeding timing.

### Larval physiology in relation to altitude

#### Routine metabolic rate

There was a significant interaction between mountain and altitude, which complicated interpretation of RMR. Posthoc tests indicated no significant differences in RMR between tadpoles sampled from high and low sites on IME or LAW. In regards to the other three mountains, there were significant differences but not always in the same direction: for DUB and MNT tadpoles sampled from high elevation showed a decreased RMR relative to those from low elevation, but the largest difference due to elevation was at LOM, where tadpoles from high elevation showed a substantial increase in RMR compared to those from low elevation (Figure 3; Table 2). However, due to the moderate power of the model (power = 0.69), it is possible that some significant relationships were missed. It has been suggested that a lower resting metabolic rate can allow more energy to be allocated to growth in resource-limited environments [16] and a link between lower RMR and increased growth rate has been found in the southern toad [29], Sydney rock oyster [30] and snapping turtle [31]. Furthermore, Sears [6] found that both increased growth rates and reduced RMR were positively correlated with altitude in sagebrush lizards. The three mountains in our study system where RMR was lower in individuals from high-altitudes (although the difference was very small for individuals from LAW) have been shown to be locally adapted to temperature parameters, with larval period decreasing and growth rate increasing at high-altitude [40]. Therefore, the lower RMR of individuals from high-altitude from DUB, MNT and LAW, is in line with the increased growth rates observed at these sites. Lindgren and Laurila [16] did not find a link between growth rates and RMR in *R. temporaria* along a latitudinal gradient in Sweden. Therefore, this is the first tentative evidence of reduced RMR being linked to increased growth rate as an adaptation to low-temperatures in an amphibian. However, the significantly higher RMR at high- than low-altitude in LOM (Figure 3), and the non-significant difference between LAWHIGH and LAWLOW in terms of RMR, could suggest that local conditions other than altitude are also important in driving divergence in RMR. For instance, the lowest RMR were observed in individuals from IMELOW and LOMLOW, where spawning occurred at the lowest temperatures of all the sites (apart from LAWHIGH), and this could suggest that low RMR is beneficial in tolerating low temperatures at all altitudes, particularly early in the season when temperatures can fall after spawning. It has also been speculated that increased growth rate can be a result of increased time spent foraging, facilitated by lower predator presence in low-temperature environments, and is unrelated to RMR [52-54]. Furthermore, RMR is influenced by temperature and there was up to a 3°C difference in temperature whilst measuring RMR of individuals from different sites, as well as a difference between the temperature at which the tadpoles were raised (15°C) and the temperature at which RMR was measured (19-22°C). Therefore, further research is needed to assess whether site-specific differences in RMR remain constant through time and whether other mountains with breeding sites of above 900 m also show reduced metabolic rate at high- vs. low-altitude.

#### Freeze tolerance

Just over half of the tadpoles that were frozen survived (61%; Table 2) and altitude was significant in predicting freeze survival: individuals sampled from low-altitude survived freezing significantly better than those from high-altitude (z = 4.20, p < 0.01). Voituron *et al.*[3] suggested that *R. temporaria* adults were freeze intolerant, as 100% mortality was observed after eight hours of complete bodily fluid freezing. However, Pasanen and Karhapää [55] found that *R. temporaria* adults could survive 24 hours in a sub-zero environment but died within three days (the actual period an individual was frozen was not measured in that study). Our results suggest that tadpoles of *R. temporaria* are also capable of surviving short periods of freezing. This is the first time, to the best of our knowledge, that larval freeze tolerance has been demonstrated in any amphibian.

Although the results presented here suggest that tadpoles are capable of surviving being frozen, the finding of a greater survival of low- compared to high-altitude individuals appears counterintuitive, given the longer period of sub-zero temperatures at high- than low-altitude in Scotland [33] and the sub-zero average winter temperature of -2.2°C at high-altitude, compared to 1.0°C at low-altitude at the breeding sites used in this study [45]. Indeed an increase in freeze tolerance with altitude has been found in plants (*Arabidopsis thaliana*[56]) and insects [18,57], but freeze tolerance and altitude has not been explicitly linked in herptiles. However, evolution of freeze tolerance in frogs, lizards and turtles has been linked to ecological pressures relating to winter temperatures experienced [35,58,59]. In general, freeze tolerant species are those that terrestrially overwinter in sub-zero temperatures, as opposed to avoiding freezing in deep water bodies [60]. Therefore, a higher freeze tolerance would suggest that low-altitude tadpoles are more often exposed to freezing temperatures, whereas high-altitude tadpoles may avoid such exposure altogether by inhabiting deep water bodies or metamorphosing prior to winter. Formation of deep water pools is inhibited at high-altitudes in Scotland due to the rocky, exposed landscape [61]. Therefore, freeze exposure is more likely avoided in high-altitude tadpoles by metamorphosing within a single active season, facilitated by a faster growth rate in conjunction with a lower RMR ( [45], this study). Therefore, our results potentially suggest that of the tadpoles found overwintering in Scotland [19], low-altitude individuals are more likely to overwinter as tadpoles than high-altitude individuals. However, it is possible that long periods of snow cover at high-altitude [33] could insulate tadpoles from freezing temperatures even in shallow water bodies. Therefore, further field research is needed to assess whether fewer, if any, high- than low-altitude individuals overwinter as tadpoles.

## Conclusion

*R. temporaria* adults do not show behavioural adaptations in terms of breeding at lower temperatures, instead they delay spawning based on the temperature experienced. However, *R. temporaria* larvae appear to have the potential to physiologically adapt to low-temperature environments, although these relationships, and the results we obtained for routine metabolic rate and freeze tolerance, are not always straightforward to interpret. Therefore, our results suggest that survival at high-altitude may be facilitated by physiological mechanisms that permit faster growth rates, allowing completion of larval development within a shorter period of time, alleviating the need for adaptations that extend the time available for larval growth. How individuals respond to environmental temperature at a local level is an important step in relating ecological and evolutionary pressures to phenotypes.

## Methods

### Adult spawning behaviour in relation to altitude

#### Data collection

Paired high- (above 700 m; *R. temporaria* occur to over 1000 m in Scotland [43]) and low-altitude (below 300 m) sites from five mountains within west central Scotland were selected for study (Table 3). The study sites have low neutral genetic population structuring (high between-site gene flow) and show local adaptation of larval traits in relation to temperature [40,45]. Air temperatures were recorded every two hours at each site between March 2010 and October 2011 using Thermocron i-buttons (Dallas Semiconductor/Maxim, London) and downloaded to a laptop every six months using a USB i-button adapter (Dallas Semiconductor/Maxim, London) and the software, Thermodata viewer (Thermodata pty Ltd., Melbourne) (as per [40,45]). Sites were visited from early February 2011 and date of spawning was recorded as the day egg masses were first observed at each site. The daily mean temperature on the day of egg mass observation was calculated for all sites. Although the majority of egg masses were at or below Gosner stage 10 on this date, and thus likely to have been laid no more than 100 hours previously [62], it is possible that spawning activity started prior to this. Therefore, the daily mean temperatures and the overall average for the week prior to egg mass observation were also calculated. In addition, degree days to egg observation were calculated for each site (a statistic commonly used to predict flowering date in plants; [63]), using the following approach: From the 1^st^ January 2011, degrees above the threshold for development (set at 5°C) were calculated per day using the formula: ((daily maximum temperature + daily minimum temperature)/2)-threshold value. The resulting values were summed to give the total degree days [63].

#### Statistical analyses

Linear regression models were used to assess whether the temperature and date at which individuals spawned had a significant relationship with altitude (continuous variable; m) using: 1) the date parameter, Julian day; and 2) the temperature parameters: mean temperature on the date spawn were observed, the mean temperature for the week prior to spawn observation, and degree days over the threshold 5°C.

### Larval physiology in relation to altitude

#### Sampling

Ten *R. temporaria* egg masses were collected soon after laying (Gosner stage 10 or below; [64]) from each of the sites monitored for adult breeding phenology. Egg masses were collected during the 2011 breeding season, transferred to the laboratory and maintained in individual sterilised water tanks until hatching at 10°C. At hatching (Gosner stage 22), a randomly selected subset of ten tadpoles were removed from each egg mass and placed in groups of five in two individual 1.3 L plastic baskets with a 0.1 cm mesh. Baskets were placed in large tanks in a common 15°C treatment room. Water quality was maintained using a flow-through system and tadpoles were fed *ad libitum* with a 1:2 mixture of finely ground dried fish and rabbit food (for further details see [45]).

#### Routine metabolic rate

Tadpoles were allowed to develop until hind leg toe differentiation became apparent, in the early stages of metamorphosis (Gosner stages 36–39). Twenty individuals per site (one individual per basket*two baskets per family*ten families per site) were transferred to individual containers and allowed to acclimatise to laboratory conditions in 100% oxygenated water for an hour prior to commencement of experimental procedures. After this period, tadpoles were moved into 8 ml respiration tubes filled with 100% oxygenated water and the lids immediately sealed. Tubes were placed in a dim, quiet location to reduce disturbance during the experiment. Respiration tubes remained closed for one hour. At the end of this period, the lid was removed and the oxygen saturation of the water was measured using an oxygen meter and probe (Strathkelvin Instruments, UK). The same procedure was carried out using a control tube containing no tadpole, to account for any oxygen consumption caused by microbial action. The oxygen meter was calibrated prior to each use using 100% and 0% oxygenated distilled water as standards. Distilled water was fully oxygenated using an aquatic bubbler and fully deoxygenated by adding sodium sulphite [65]. The oxygen probe was maintained at a constant temperature to avoid biases caused by thermal fluctuation using a flowing water bath. The temperature of the water bath was monitored throughout using a submerged thermometer. Once the experiment was completed, tadpoles were immediately blotted dry to remove excess water, weighed using an electric balance (to the nearest 0.1 g) to account for size differences among individuals in metabolic rate calculations, and returned to 100% oxygenated water. Although tadpole activity levels were very low whilst sealed in the respiration tubes, some short bursts of spontaneous activity were observed. Therefore, the metabolic rate estimates are considered as routine, which includes resting metabolic rate plus any extra energy expenditure due to spontaneous activity and stress [16]. Percentage of oxygen consumed was calculated by subtracting the oxygen saturation of the control tube (i.e. the oxygen used by microbial activity) from the oxygen saturation of each respiration tube (i.e. the total oxygen consumed by both tadpole and bacterial activity). Percentage saturation was converted to ml l^-1^ using standard conversion tables based on water temperature during the experiment (water temperature varied between 19-22°C depending on the date of the experiment). Routine metabolic rate (RMR) was calculated for each individual as millilitres of oxygen consumed per gram weight per hour (ml O_2_ g^-1^ h^-1^).

#### Freeze tolerance

Ten individuals per site (one individual per family*ten families per site; different individuals to those used in the RMR experiment) were moved to individual containers at Gosner stage 36–39 (hind leg toe differentiation) and deprived of food for 48 hours. Tadpoles were sealed within individual containers containing 80 ml of water and cooled to 4°C for 24 hours to cause inactivity. Containers were then gradually cooled, over a period of six hours, just until all the water in the container became completely frozen. Following this, tanks were gradually warmed (over a period of 14 hours) to 15°C and this temperature was maintained for one hour. All tadpoles were assessed for normal swimming behaviour at this point and the number of individuals that were still alive and exhibiting normal behaviour were recorded as the measure of freeze survival.

#### Statistical analyses

To evaluate whether RMR or freeze survival varied by altitude (fixed factor of interest; considered as a categorical variable of low or high) a generalised linear mixed model approach (GLMM) was used, as implemented in R v2.12.1 [66] using the lme4 package [67]. Mountain was included as a random factor in all models and family was nested within altitude in the RMR model, but multiple individuals from the same egg mass were not used in the freeze tolerance experiments thus family was not included in the freeze tolerance model. Weight was also included as a linear covariate in the freeze survival models, but was already accounted for in the measurement of RMR (ml O_2_ g^-1^ h^-1^). For RMR the model was assessed under a normal distribution and for freeze survival a binomial distribution was used. Each model parameter and interaction was sequentially removed from the models and a likelihood ratio test used to evaluate parameter significance. Only parameters that significantly changed the log likelihood when removed from the model were included in the final model. Significant differences due to the model parameters that showed interactions were evaluated using a Tukey’s HSD test [68], which works in conjunction with an ANOVA of the GLMM. A post-hoc power analysis of the ANOVA was carried out for RMR using G*Power v3.1.3 [69]; effect size was calculated using the formula in Krebs [70].

### Animal ethics statement

All the protocols used in this study were approved by the U.K. Home Office: Project License 60/4110.

## Availability of supporting data

The data sets supporting the results of this article are available in the Dryad repository, doi:10.5061/dryad.ks2j1, https://datadryad.org/resource/doi:10.5061/dryad.ks2jl[71].

## Competing interests

The authors declare that they have no competing interests.

## Authors’ contributions

This research forms part of APM’s PhD thesis work on the population genetics of *R. temporaria* in Scotland with BKM and RB; she was responsible for all aspects of the work, from experimental design, sampling, experimental procedures, analyses and writing. BKM contributed to experimental design, advice on analyses, and editorial content. RB contributed to editorial content. All authors read and approved the final manuscript.

## Supplementary Material

Additional file 1 **The results of the likelihood ratio test for the freeze tolerance data.** Removal of mountain from the model did not significantly change the log likelihood (Models 4 and 8) and reducing the complexity of the model from Altitude*Weight to Altitude + Weight did not significantly affect the log likelihood (Models 5 and 8). Therefore, Model 8 was chosen as the final model and used to run the GLMM. Click here for file
