# Supplementary material for: Behavioural and physiological adaptations to low-temperature environments in the common frog, Rana temporaria
Source: BMC Evol Biol. 2014 May 23;14:110. doi: 10.1186/1471-2148-14-110 (PMC4037278; doi:10.1186/1471-2148-14-110)
Supplement: Additional file 1 — The results of the likelihood ratio test for the freeze tolerance data. Removal of mountain from the model did not significantly change the log likelihood (Models 4 and 8) and reducing the complexity of the model from Altitude*Weight to Altitude + Weight did not significantly affect the log likelihood (Models 5 and 8). Therefore, Model 8 was chosen as the final model and used to run the GLMM. [file 1471-2148-14-110-S1.docx]

**The results of the likelihood ratio test for the freeze tolerance data.** Removal of mountain from the model did not significantly change the log likelihood (Models 4 and 8) and reducing the complexity of the model from Altitude*Weight to Altitude+Weight did not significantly affect the log likelihood (Models 5 and 8). Therefore, Model 8 was chosen as the final model and used to run the GLMM.

Model 1: freeze$Survival ~ freeze$Mountain * freeze$Altitude * freeze$Weight

Model 2: freeze$Survival ~ freeze$Mountain * freeze$Weight

Model 3: freeze$Survival ~ freeze$Mountain * freeze$Altitude

Model 4: freeze$Survival ~ freeze$Altitude * freeze$Weight

Model 5: freeze$Survival ~ freeze$Mountain + freeze$Altitude + freeze$Weight

Model 6: freeze$Survival ~ freeze$Mountain + freeze$Weight

Model 7: freeze$Survival ~ freeze$Mountain + freeze$Altitude

Model 8: freeze$Survival ~ freeze$Altitude + freeze$Weight

Model 9: freeze$Survival ~ freeze$Mountain

Model 10: freeze$Survival ~ freeze$Altitude

Model 11: freeze$Survival ~ freeze$Weight

#Df LogLik Df Chisq Pr(>Chisq)

1 15 -32.938

2 8 -48.055 -7 30.2340 8.602e-05 ***

3 8 -38.715 0 18.6807 < 2.2e-16 ***

4 4 -40.077 -4 2.7254 0.6047840

5 6 -37.633 2 4.8878 0.0868218 .

6 5 -49.966 -1 24.6656 6.819e-07 ***

7 5 -38.890 0 22.1521 < 2.2e-16 ***

8 3 -40.324 -2 2.8685 0.2382981

9 4 -49.966 1 19.2836 1.127e-05 ***

10 2 -40.895 -2 18.1430 0.0001149 ***

11 2 -51.462 0 21.1354 < 2.2e-16 ***

---

Signif. codes: 0 ‘***’ 0.001 ‘**’ 0.01 ‘*’ 0.05 ‘.’ 0.1 ‘ ’ 1
